# Lower 24-Month Relative Survival among Black Patients with Non- Hodgkin’s Lymphoma: An Analysis of the SEER Data 1997–2015

**Published:** 2021

**Authors:** Maria J Nieto, Zhen Li, Hasan Rehman, Muhammad Wasif Saif

**Affiliations:** 1 Northwell Health Cancer Institute & Donald and Barbara Zucker School of Medicine at Hofstra, Lake Success, NY, USA; 2 Wyckoff Medical Center, New York, NY, USA

**Keywords:** SEER, non-Hodgkin lymphoma, Lymphoma, Rituximab, CHOP regimen, RCHOP, Diffuse large B cell lymphoma, Racial disparities

## Abstract

**Background:**

Recent progress in the therapies used in patients with Non- Hodgkin’s lymphoma has improved survival. The incidence has been reported to be decreasing in the last few years, accounting for 4% of all cancers. This study analyzed time trends for incidence, mortality, and prevalence of NHL.

**Methods:**

We analyzed the SEER Cancer Database from 1997 to 2015. Join point regression analysis was used to determine age-adjusted incidence rates, 24-month relative survival rate, and to identify racial/ethnic groups with a lower survival.

**Results:**

The trend in incidence of NHL decreased between 2008 and 2011 at an annual percentage change rate of 3.74%. The male predominance among NHL patients between 1997–2015 was 57%. The number of male patients affected with NHL has been similar in the last 20 years. Female predominance with NHL was higher in 1998 at 46 %, and lower in 2010 at 42.85%. The 24-month relative survival rate was higher among white patients as compared to black patients with NHL.

**Conclusions:**

Our analysis demonstrated that the incidence of Non-Hodgkin’s Lymphoma has decreased among minorities; however, the outcomes are inferior in terms of survival. This analysis showed an inferior 24-month relative survival rate among black patients compared with white patients. This analysis demonstrates the need for further research in NHL to determine the biological differences and social factors that influence the lower survival among black patients with NHL.

## Introduction

Recent progress in the therapies used for patients with Non- Hodgkin’s lymphoma (NHL) has improved survival. In 2020, 77,240 people were diagnosed with NHL. Although it accounts for 4% of all cancers, the incidence has been reported to be decreasing in the last few years. About 26% of people will expire from NHL (15% males and 11% females) [1].

Non-Hodgkin lymphoma arises from the clonal expansion of B, T, and natural killer (NK) cells. There is a significant degree of heterogeneity in NHL and this is likely related to different degrees of differentiation and maturation of these cells [2]. These hematological malignancies exhibit different tumor behavior and are responsive to different chemotherapy agents which impacts clinical outcomes. There are patients who can be cured with current regimens; however, subtypes such as indolent and some aggressive lymphomas remain incurable necessitating treatment with new therapies including immunotherapy, targeted therapy, CAR T cells, and hematopoietic stem cell transplant.

One of the strongest risk factors for NHL is severe immunosuppression either secondary to HIV/AIDS or organ transplantation. The incidence of HIV infection associated lymphoma has significantly declined in the last two decades likely secondary to the use of antiretroviral therapy [3]. The HIV infection associated lymphomas include a variety of conditions including diffuse large B-cell, with extra-nodal location mostly localized to the gastrointestinal tract and central nervous system. These lymphomas tend to be aggressive and may have an association with Epstein-Barr virus [4]. There are also inherited disorders that have been associated with NHL including common variable immunodeficiency, Wiskott-Aldrich syndrome, ataxia telangiectasia, and Nijmegen breakage syndrome which affect patients primarily with B cell lymphomas during childhood [5]. In addition, infectious diseases such as Helicobacter pylori have been linked with gastric mucosa-associated lymphoid tissue (MALT) lymphomas by initiating an immunological response. This, in turn, causes gastritis and therefore leads to the formation of lymphoid follicles within the stomach [6]. Individuals with Human T-lymphotropic virus type I can develop an overly aggressive adult T-cell leukemia/lymphoma [7]. Patients with hepatitis C associated B cell lymphoma (splenic marginal zone lymphoma) have demonstrated regression of the lymphoma with antiviral treatments [8,9]. Other marginal zone lymphomas have been associated with Chlamydia psittasi in the ocular adnexa, and Borrelia burgdorferi in the skin [10,11]. Exposure to radiation and/or chemotherapy has also shown to have an increased risk of developing lymphoma [12].

The 5-year overall survival rate for patients with NHL is 72%. Non-Hodgkin’s lymphoma is a systemic disease in 80%–85% of patients. There is a different biological behavior among subtypes of B cell lymphomas, which correlates directly with survival. Patients with NHL benefit from a multi-disciplinary approach. Cytotoxic chemotherapy is still an important component for the treatment for patients with NHL. Patients with limited and localized stage I and II disease can be treated with external-beam radiation therapy [13]. There has been a shift in the treatment of NHL, with new strategies being used to amplify the immune response. One of the therapies that has demonstrated the benefit of targeting the immune response is allogeneic stem cell transplantation. The donor provides healthy stem cells; T cells from the donor target the malignant cells from the recipient and produce a therapeutic graft versus tumor effect. Vaccinations have been studied for several years as well and have demonstrated that they can induce an immunologic and clinical response. Monoclonal antibodies have also made a significant improvement in overall survival, targeting multiple antigens including CD20, CD52, and CD40. These agents bind to the B-cell surface which leads to an increase in complement-dependent cytotoxicity. Lastly, immune check point inhibitors have also been tested in NHL. These drugs have proven to be efficacious for some of these malignancies by targeting CTLA-4, PD-1, and PD-L1.

Despite the advances in the treatment of Non-Hodgkin’s Lymphoma in recent years, there are disparities in outcomes based on race, ethnicity, and other areas that affect the management of these patients. Recognition of these inequalities will lead to further investigation of pathways to improve the care and management of all individuals.

This retrospective study was designed to analyze time trends for incidence, age, sex, race, stage, mortality, 24-month relative survival rate, and prevalence of NHL. We analyzed the SEER data between 1997 to 2015 with the objective of identifying prognostic factors that may increase or reduce survival.

## Methodology

We used the Surveillance, Epidemiologic, and End Results (SEER) database to determine the incidence of NHL over the last 18 years. In addition, we sought to determine age, race, sex, and stage-based differences in the incidence and survival of NHL between 1997–2015. The SEER Program of the National Cancer Institute annually collects cancer incidence and survival data from 9 population-based cancer registries across the United States. Non-Hodgkin’s lymphoma tumors (site codes, ICD-0-3, C85.90 to C85.99, and C82.90) were obtained from the SEER database from 1997 through 2015. Histologic codes 9590, and 9670 to 9720 were identified as NHL.

We used the join point regression model to analyze the data. Join point regression model is a trend analysis software developed by the National Cancer Institute for the analysis of data from the Surveillance Epidemiology and End Results Program. This program describes changes in the data trends by connecting several different line segments at join points. The program starts with the minimum number of join points (e.g., 0 join points equates to a straight line) and tests whether more join points are statistically significant and must be added to the model (up to that maximum number). This enables the user to test if an apparent change in trend is statistically significant. The tests of significance use a Monte Carlo Permutation method. Once the number of join points has been obtained, the different models with join points are compared by estimating their Bayesian Information Criterion (BIC).

## Results

**Figure 1:**
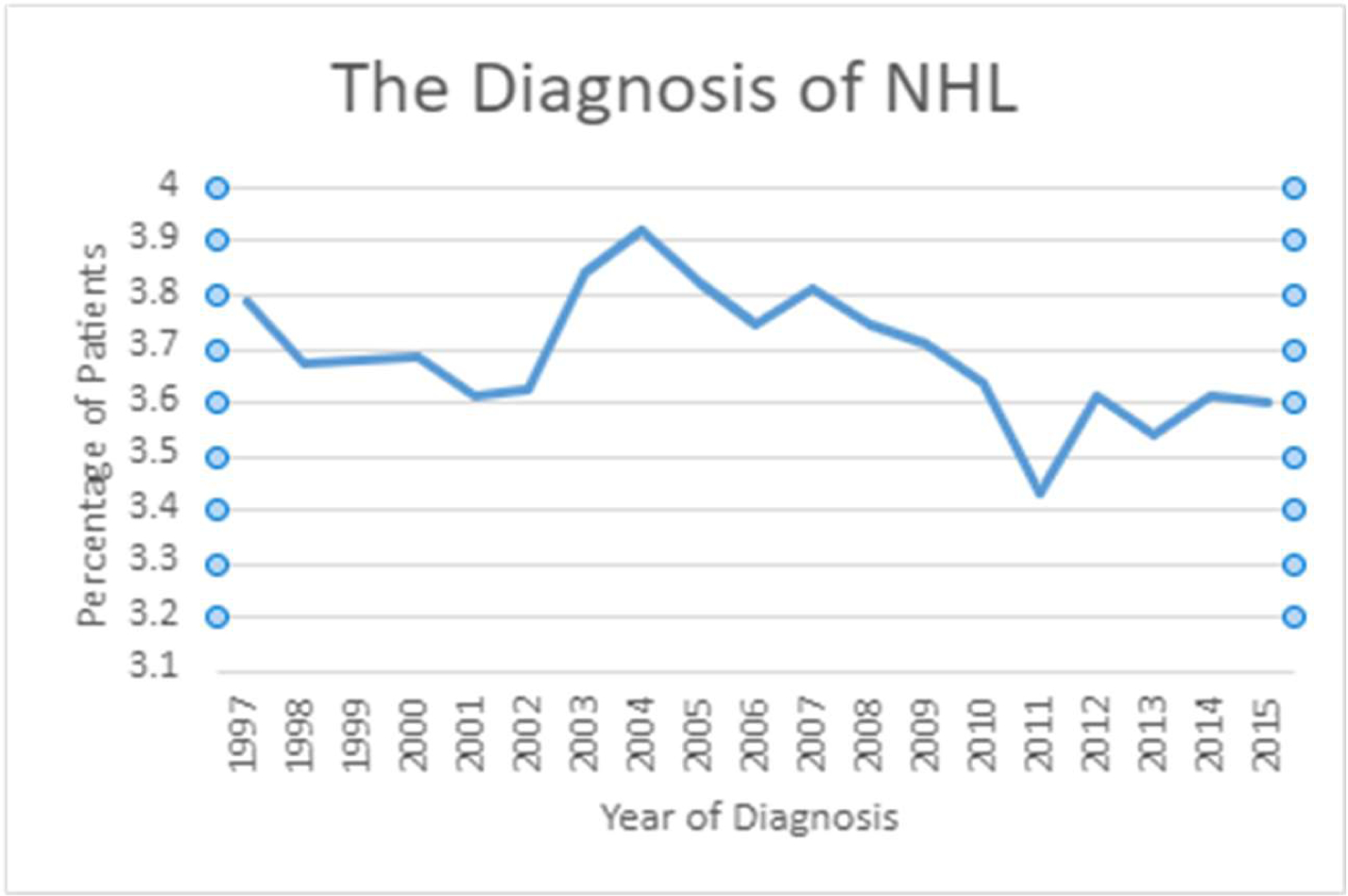
The Diagnosis of Non-Hodgkin’s Lymphoma between 1997–2015.

The characteristics of patients with Non-Hodgkin’s Lymphoma as outlined in the SEER database from 1997–2015 are summarized in Table 1. The diagnosis of NHL increased in 2004 by 3.9% and dropped to 3.4% in 2011 (Figure 1).

**Figure 2:**
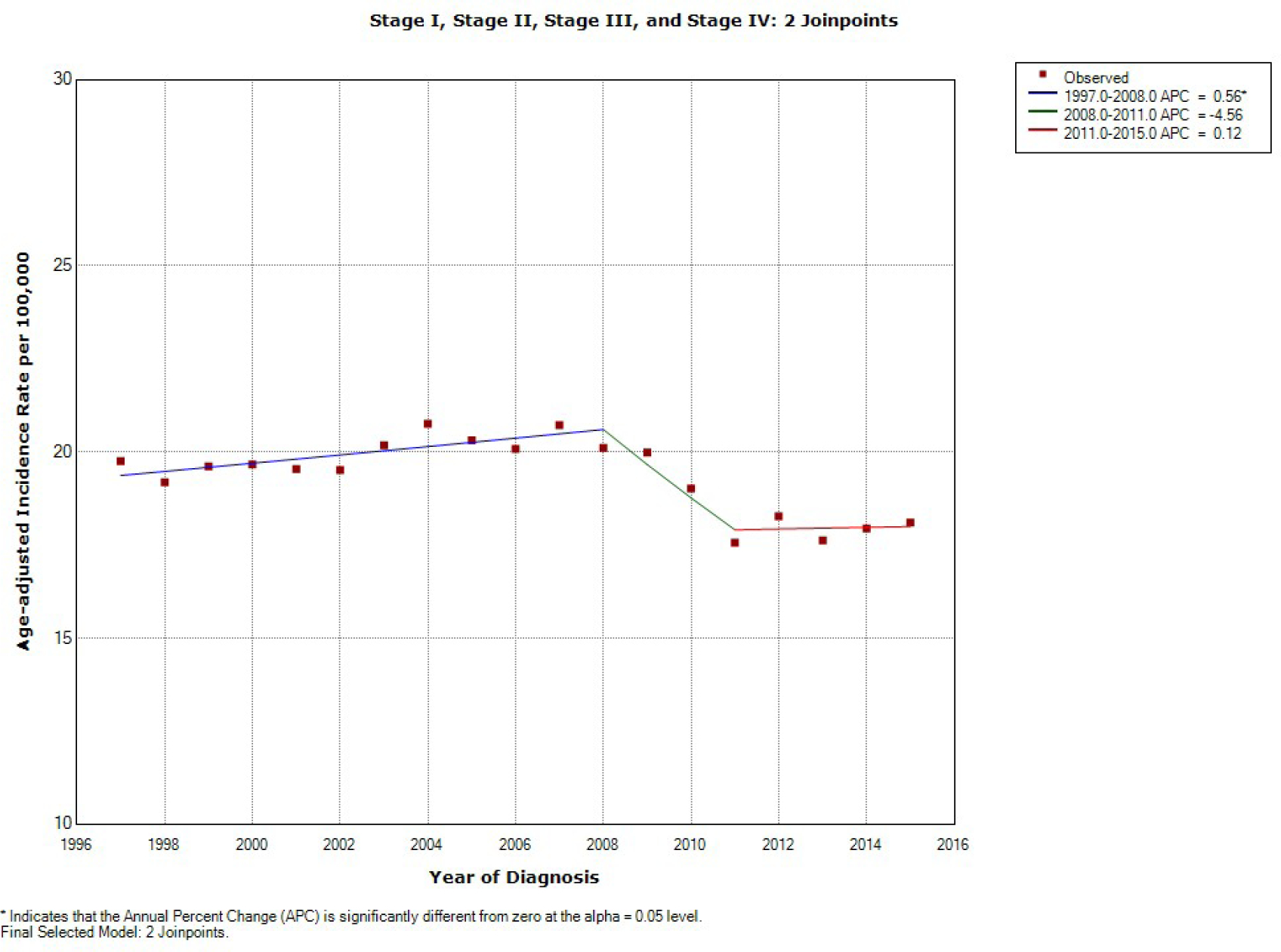
Join point regression of diagnosis of Non-Hodgkin’s Lymphoma, by year. (*) P .05. APC, annual percentage change.

The age-adjusted incidence rate per 100,000 population of NHL is described in Figure 2. Our results indicate that the absolute incidence of NHL increased at an annual rate of 0.56% until 2008 which was statistically significant. It decreased between 2008 and 2011 at an annual percentage change rate of 4.56% which was not statistically significant. It had an upward trend between 2011 and 2015 at an annual percentage change rate of 0.12% which was also not statistically significant.

### Age

**Figure 3:**
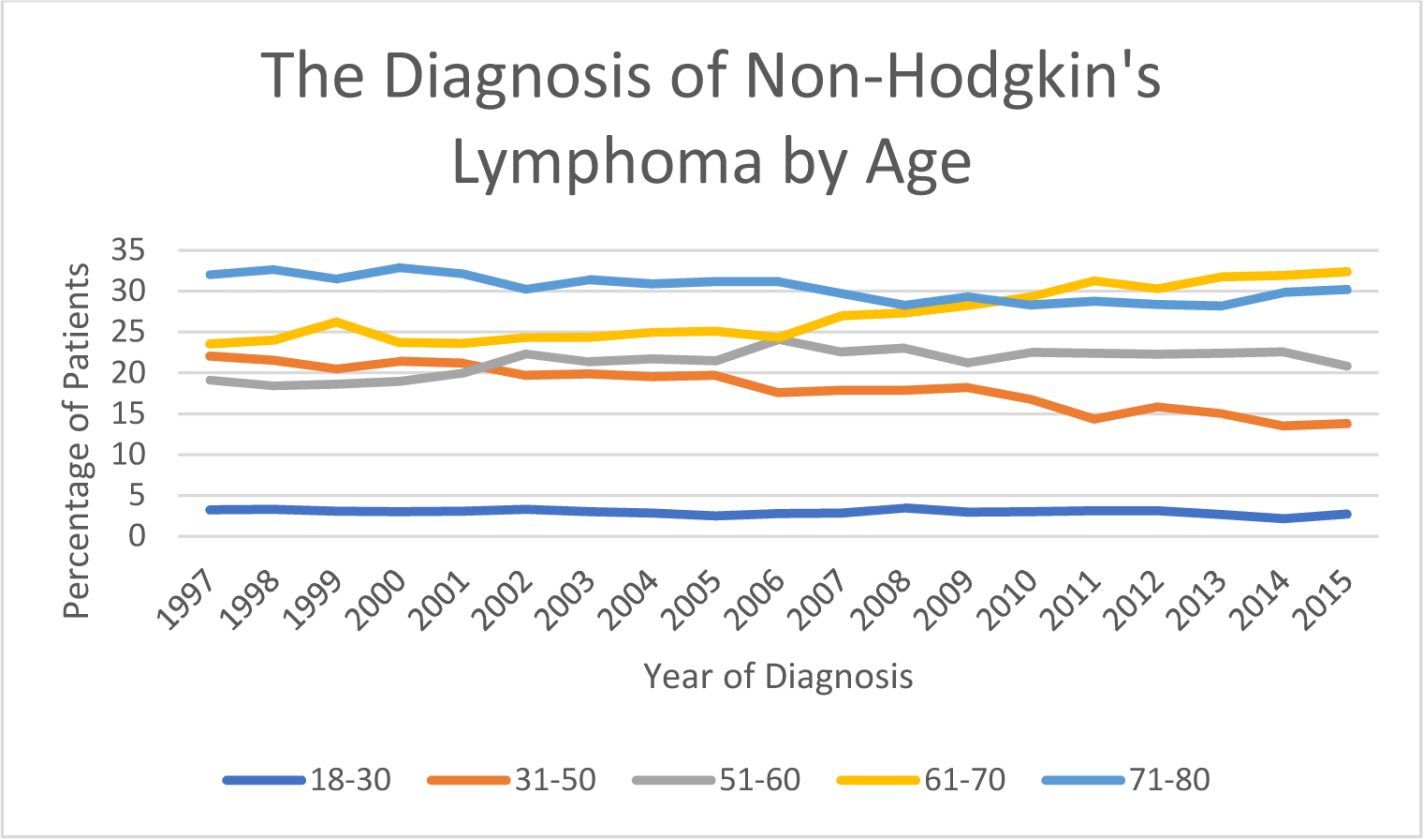
The Diagnosis of Non-Hodgkin’s Lymphoma by Age.

Figure 3 reveals that patients aged 18–30 represented less than 5% of the total percentage of patients. The analysis also showed that the incidence of patients between ages 31–50 has been progressively decreasing from 22.04 % in 1997 to 13.79% in 2015. The incidence of patient ages between 51–60 was 19.17% in 1997, which rose to 24.11% in 2006 and decreased to 20.85% in 2015. The major increase was observed in patient ages between 61–70 who initially had an incidence of 23.56% and rose to 32.35% in 2015. Elderly patients ages 71–80 showed an incidence of 32.02% in 1997. There was a moderate decrease to 28.16 % in 2013; however, incidence rose again to 30.35% in 2015.

### Sex and stage

**Figure 4:**
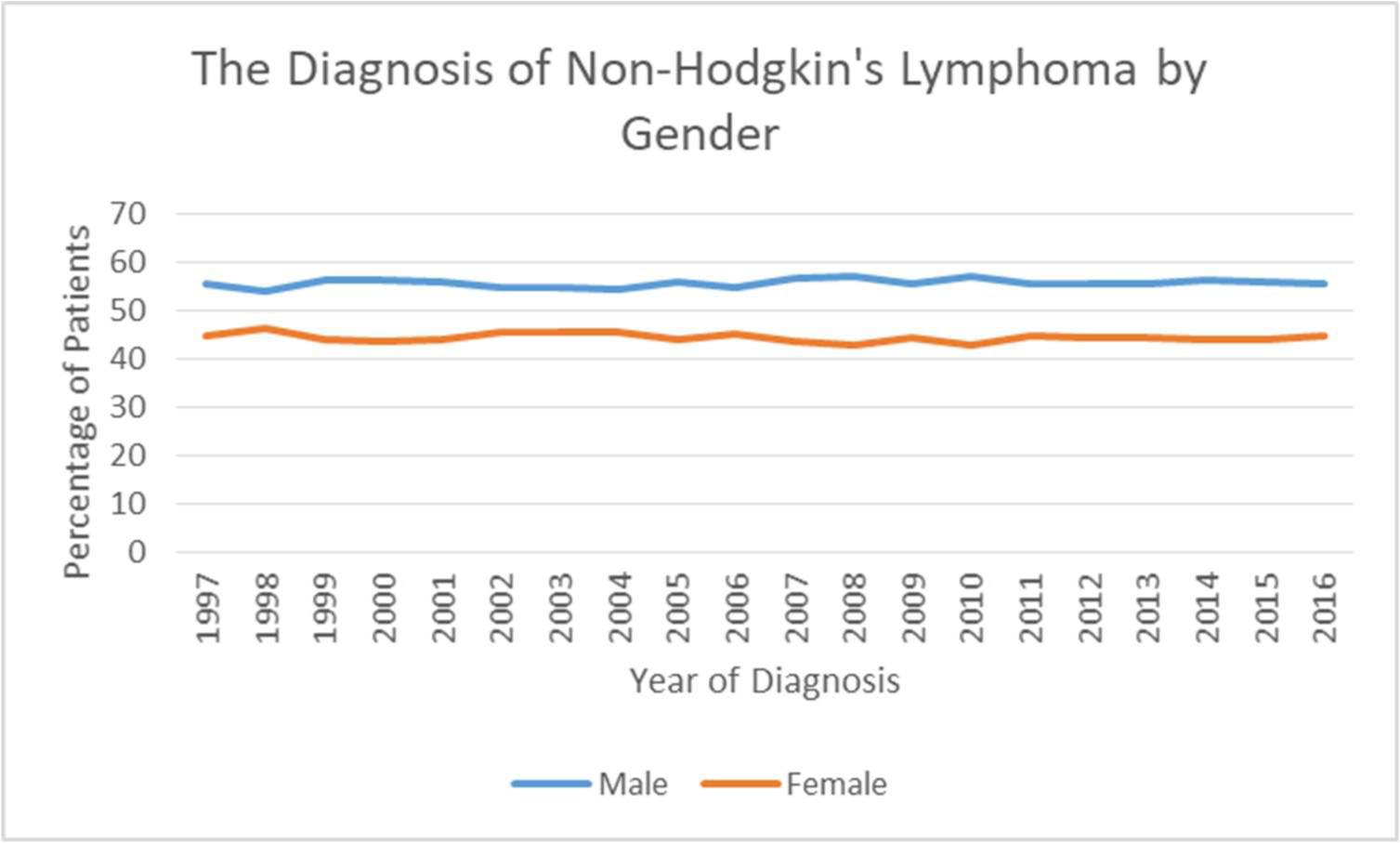
The Diagnosis of Non-Hodgkin’s Lymphoma by Gender.

**Figure 5:**
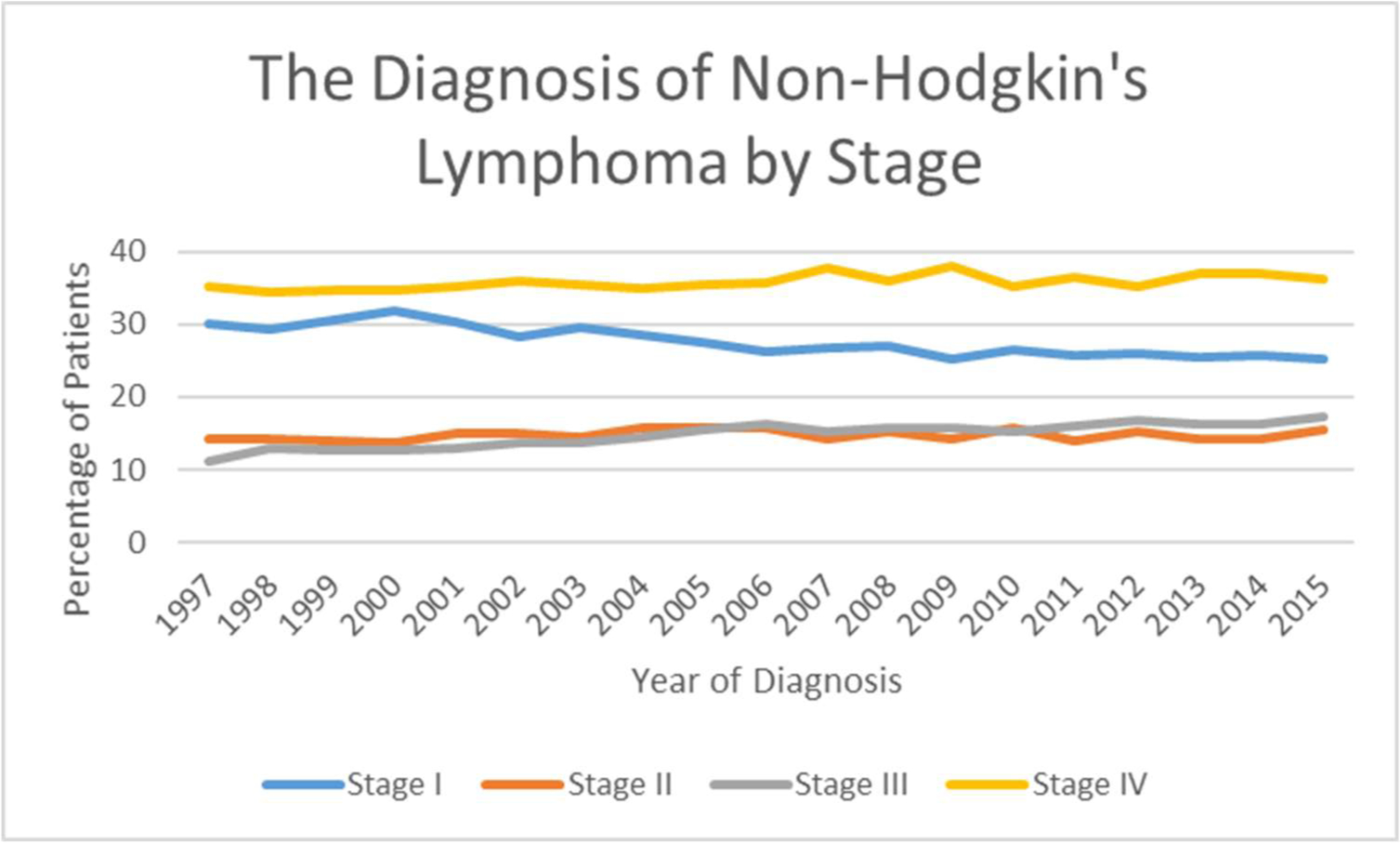
The Diagnosis of Non-Hodgkin’s Lymphoma by Stage.

Figure 4 reveals that the predominance of NHL patients between 1997–2015 were male accounting for 57% in 2010. The percentage of male patients has been similar in the last 20 years. The percentage of female patients with NHL was higher in 1998 at 46% but decreased to 42.80% in 2010. Figure 5 reveals that the percentage of stage I NHL increased to 32% by 2000 and decreased to 25% by 2015. Stage II disease increased to 16% in 2004 and then decreased to 14% in 2014. Stage III increased from 11% in 1997 to 17% in 2015. Stage IV increased from 35% in 1997 to 37% in 2007, but then decreased to 36% in 2015.

### Race

**Figure 6:**
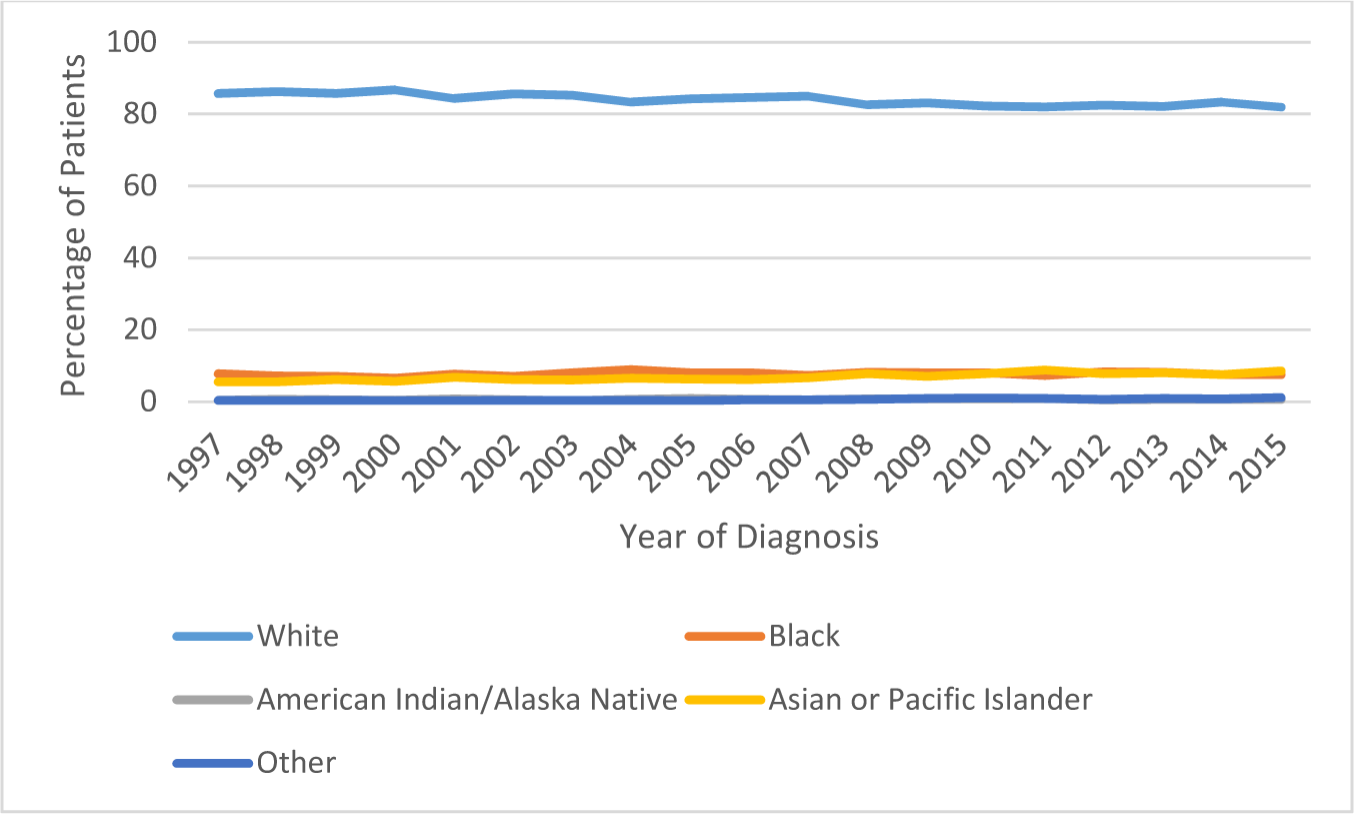
The Diagnosis of Non-Hodgkin’s Lymphoma by Race.

The percentage of white patients decreased between 1997 to 2015 from 85.7% to 82% as seen in Figure 6. The percentage of black patients increased from 7.8% in 1997 to 9% in 2004. This dropped again to 7.6% in 2015. The percentage of American Indian patients increased from 0.42% in 1997 to 0.89% in 2011 and further dropped to 0.71% in 2015. The percentage of Asian patients increased from 5.58% in 1997 to 8.6% in 2015.

**Figure 7:**
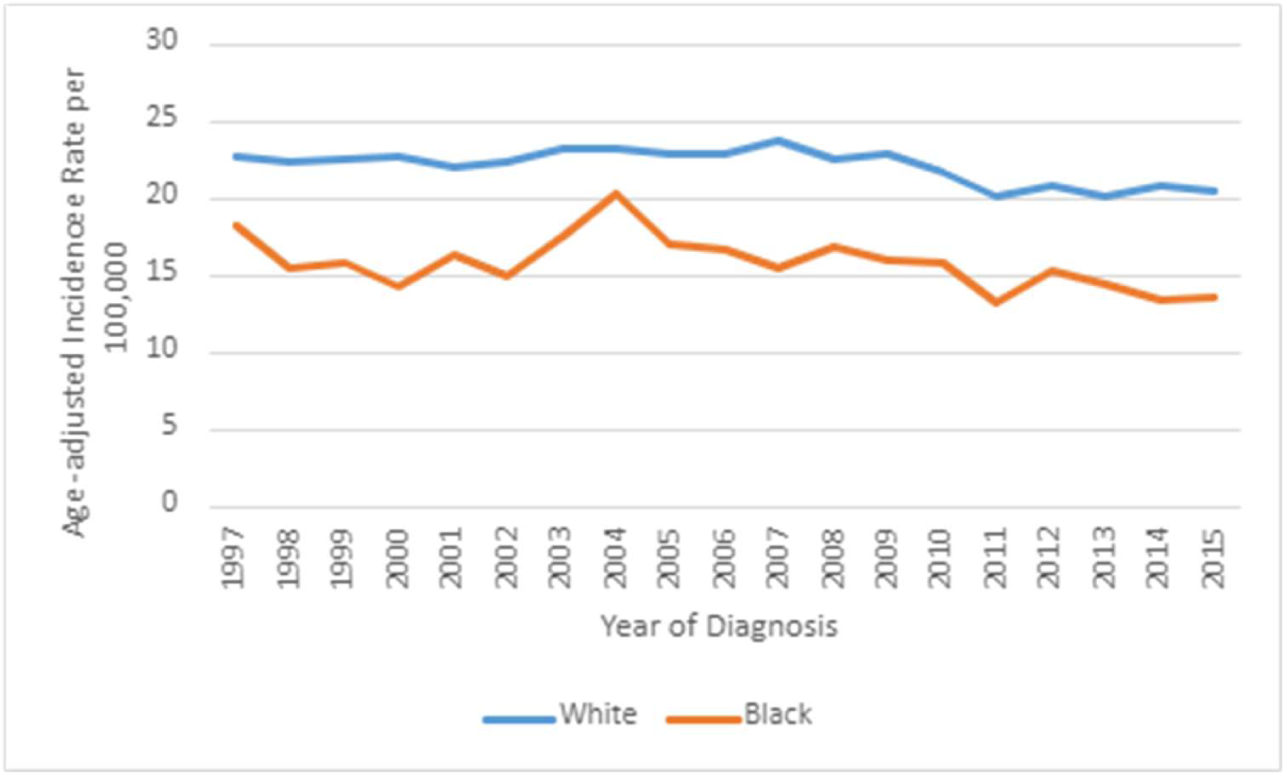
Age-adjusted Incidence Rate per 100,000 population by Race.

Figure 7 reveals the age-adjusted incidence rate by race was higher among whites; the incidence peaked in 2007 to a rate of 23.81 per 100, 000. The Age-adjusted incidence rate for black patients peaked in 2004 to a rate of 20.37 per 100,000.

### Survival among Whites compare to Blacks

**Figure 8:**
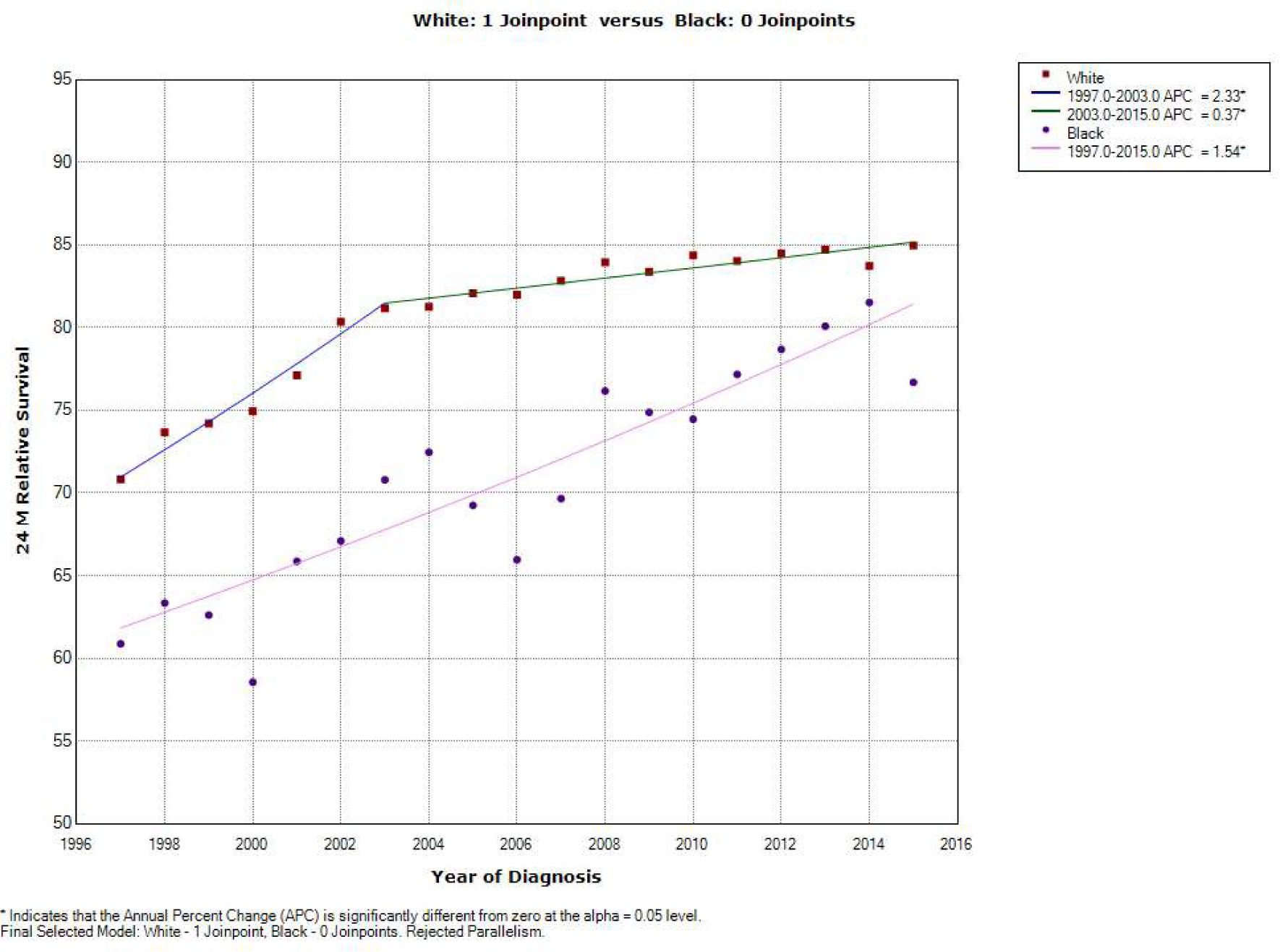
Join point regression of 24 -month Relative Survival Rate by Race, by year. (*) P .05. APC, annual percentage change.

Figure 8 reveals an increase in the 24 -month relative survival rate in whites, at an annual percentage rate of 2.33% between 1997 and 2003 that was statistically significant. There was also an additional increase in the 24-month relative survival rate in white patients at an annual rate of 0.37% between 2013 and 2015. In contrast, the 24-month relative survival rate for black patients did not have a join point in the regression analysis. The 24-month relative rate in black patients between 1997 and 2015 only increased by 1.54% which was statistically significant.

### Age-Adjusted mortality rate among Whites compare to Blacks

**Figure 9:**
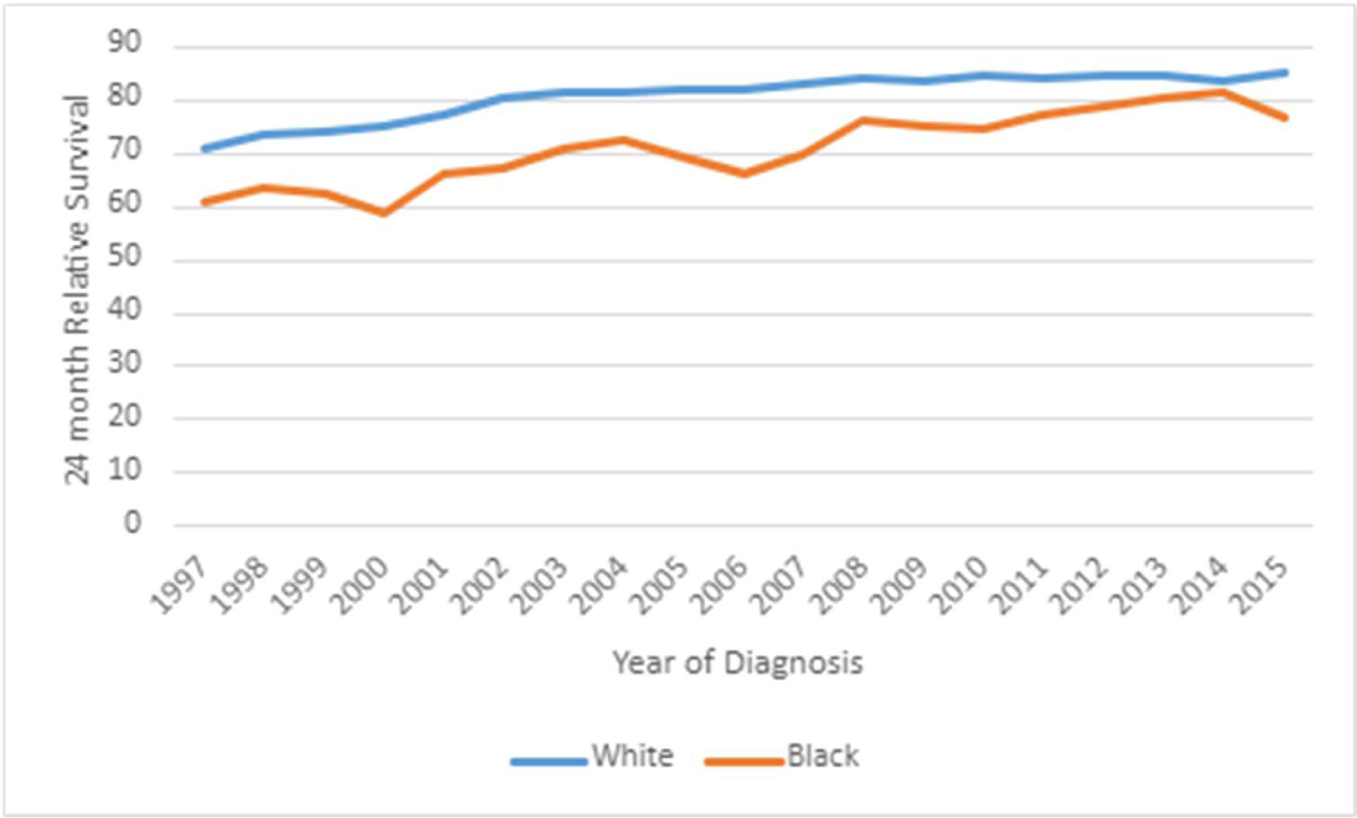
Age-adjusted mortality rate per 100,000 population by Race.

Figure 9 depicts the age-adjusted mortality rate per 100,000 population was higher among whites; the age-adjusted mortality rate peaked in 1997 to 11.3 per 100,000 and has been decreasing to 6.5 per 100,000. The Age-adjusted mortality rate in African Americans peaked in 1997 to 8.2 per 100,000 and has been progressively decreasing to 4.9 per 100,000 in 2015.

## Discussion

This study showed an upward trend in survival for patients with Non-Hodgkin’s lymphoma (NHL) in the last 18 years. This probably reflects the new advancements in treatments including chemotherapy, radiation therapy, new monoclonal antibodies, immunotherapy, bone marrow transplantation, and CAR T cells.

Our analysis demonstrated the incidence of NHL is lower among minorities; however, the outcomes are inferior in terms of survival. This analysis showed a statistically significant inferior 24-month relative survival rate for blacks compared to white patients. There have been previous studies with Follicular lymphoma showing that black patients most commonly present with aggressive disease and elevated Follicular Lymphoma International Prognostic Index (FLIPI) scores [14]. Similar findings have been reported with Chronic Lymphocytic Leukemia, as black patients present with high-risk prognostic markers, severe anemia, increase in beta-2 microglobulin levels, advanced Rai staging, and cytogenetic markers associated with a worse prognosis as compared to white patients [15,16].

Furthermore, a study evaluating the disparities in the infusion of chemoimmunotherapy among patients with diffuse large B cell lymphoma showed that there was a dramatic increase in the proportion of patients receiving chemoimmunotherapy between 1998 and 2004 following the approval of Rituximab in 1997 [17]. That study revealed that although the use of chemoimmunotherapy increased across all racial categories, certain populations were unlikely to receive appropriate treatments between 2001–2004. These patients included those who were uninsured or had Medicaid, as well as black patients and those with low socioeconomic status.

The etiology for these racial inequalities is uncertain, however a very similar outcome was also observed in a study published in Cutaneous T Cell Lymphoma [18]. The incidence for Mycosis Fungoides was higher among black patients with a greater predominance among black females. The 2-year relative survival rate was superior among white patients at 88% compared to black patients at 75% (p-value 0.02).

Further studies will be needed to determine causes of these disparities. Potential discrepancies may be attributable to misinterpretations, easy access to treatment, impaired patient-physician relationships, or physician biases. Epidemiological research has shown that hospitals with a high Medicaid rate had a higher 30 day and 12-month mortality rate compared with institutions with Medicare or private insurance [19]. In a study of Medicare Beneficiaries with cancer, black patients had lower 1- and 3-year survival rates secondary to advanced disease stage at the time of diagnosis and suboptimal use of surgery [20]. The lower socioeconomic status among blacks as compared to whites is another factor that can partially explain the disparities in outcomes of black cancer patients [21].

Analysis of tumor biology to advance research in cancer outcomes among minorities has been done in other malignancies such as breast, prostate and colon cancer [22]. Additional studies will be needed in NHL to determine differences in biology, molecular markers, epigenetics, and tumor associated inflammatory markers. The significance of these studies is to target specific immune microenvironment markers as a therapeutic intervention.

Improvement in outcomes of black patients with NHL and other cancers will require interventions, alternative research methodologies, health care policy changes, as well as an increase in the number of racial/ethnic minorities in the physician workforce and navigation programs.

This retrospective study has its limitations. The accuracy of the data depends on the availability of important information in the medical records. For example, specifics of radiotherapy, radiation treatment modalities, chemotherapy regimens, and use of salvage therapies is not available. Additional information, including The International Prognostic Index (IPI), presence of B symptoms, level of lactate dehydrogenase (LDH), performance status as per The Eastern Cooperative Oncology Group (ECOG), and number of extranodal sites is also not available. Other missing data includes information about cellular factors that has prognostic significance. For example, an unmutated germinal center gene expression pattern has a favorable prognosis compared to non-germinal center tumors with mutated immunoglobulin genes. Also, the presence of other important molecular markers including expression of BCL2, BCL6, c-myc, and TP53 were not reported.

In conclusion, our study demonstrates the need for further research in NHL to determine the biological differences and social factors that influence the lower survival among black patients with NHL. This will require an interdisciplinary dialogue, integration of research, and increasing participation in clinical trials, thereby refining the data collection on race, ethnicity and socioeconomic status. These interventions will aim at improving the care and outcomes of minorities in the future.

## Figures and Tables

**Table 1:** Characteristic of Patients with Non-Hodgkin’s Lymphoma (N=79,179).

Characteristic	No.of Patients	%
**Gender (N=79,179)**		
Male	44,072	55.66
Female	35,107	44.34
**Stage (N=73,662)**		
Stage I	21,838	29.65
Stage II	11,692	15.87
Stage III	11,751	15.95
Stage IV	28,381	38.53
**Race (N=79,179)**		
White	66,479	83.96
Black	6,152	7.77
American Indian	523	0.66
Asian	5,522	6.97
Other	503	0.64
**Age** (**N=79,179)**		
18–30	2,325	2.94
31–50	14,326	18.1
51–60	16,982	21.44
61–70	21,533	27.2
71–80	24,013	30.32
Ethnicity (N=79,179)		
Non-Hispanic	73,948	93.4
Hispanic	5,231	6.6
